# Supplementary material for: Are carbon emissions trading and green financial instruments synergistic? -Comprehensive quantitative research based on content analysis
Source: PLoS One. 2024 Mar 7;19(3):e0298601. doi: 10.1371/journal.pone.0298601 (PMC10919601; doi:10.1371/journal.pone.0298601)
Supplement: S1 Data — (ZIP) [file pone.0298601.s001.zip › Data/地级市Z_F工作报告.zip!/╔╜╬≈/╜·│╟╩╨/└┤╫╘╣1⁄2╓┌║┼í╛┬φ┐╦╩2╛▌═°í┐_2005.docx]

各位代表：



　　现在，我代表市人民政府向大会作政府工作报告，请予审议，并请市政协委员和其他列席人员提出意见。



　　一、2004年政府工作回顾

　　过去的一年，在中共晋城市委的正确领导下，市政府认真贯彻中央、国务院和省委、省政府的一系列方针政策，积极落实科学的发展观，求真务实，开拓创新，圆满完成了市四届人大五次会议确定的主要预期目标。全市生产总值完成244.4亿元，比上年增长15%，提前一年实现“十五”目标；财政总收入完成43.1亿元，增长43.1%。其中，一般预算收入完成13.9亿元，增长34.7%；城镇居民人均可支配收入达到8097元，增长14.5%；农民人均纯收入达到3278元，增长11.5%；市区空气质量二级以上天数达到192天，超力争目标12天。

　　经济结构调整继续深化。以“四大基地”、“八大产业”建设为着力点，全力构筑全市经济的“四梁八柱”。煤炭生产集中度和技术装备水平继续提高，新建成百万吨以上矿井1个，60万吨矿井1个，30万吨矿井8个，煤炭加工转化率达到25%；阳电二期前期工作全面完成，5个国家级小水电项目进展顺利；以建设高浓度氮肥基地和发展精细化工为目标，煤化工“1830”尿素项目全面投产，天脊“4060”、晋丰“3652”等尿素项目和丹峰30万吨甲醇项目正在建设；福盛钢厂100万吨轧钢项目一期工程建成投产，开发区国际铸造园区初具规模，汉通、泫氏、金工、盛固等一批高档铸造和机械加工企业迅速崛起；阳城建瓷工业园区迅速成长，已形成2000万平方米生产能力。丝麻、饮品、旅游产业也都取得了新的进展。所有制结构调整继续深化，民营经济增幅达到23.1%，创近10年来最好水平。

　　农业和农村经济势头良好。认真贯彻中央一号文件,不折不扣地落实粮食直补、税费改革和严格的耕地保护政策,农业和农村经济呈现出好的势头。粮食总产量达到80万吨，为历史上第二个丰收年。猪、牛、羊出栏分别增长23.3%、14.9%和10.2%。完成荒山造林14.9万亩，公路绿化1852公里。完成“村村通”公路2533公里。建成79处饮水工程，解决了6.3万人的饮水困难。8家农业产业化龙头企业进入全省“百龙企业”行列，厦普赛尔公司进入国家级龙头企业行列。新建宽裕型小康乡镇4个、村131个。完成扶贫移民搬迁385户、1600人,又有8300人实现稳定脱贫。

　　城市面貌有了新的变化。泽州路南段等4段总长3.1公里的城市道路建成通车。“两河”治理一期工程全面完成，形成水面10万平方米，建成百丽园、西秀园等6个景点。城市绿化覆盖率、公共绿地率和人均公共绿地面积分别达到33.1%、29.2%和8.1平方米，成为省级园林城市。多功能综合馆、体育场、黄华街西区“城中村”改造等重点城建项目完成年度目标。

　　重点工程进展顺利。继续实施投资拉动战略，固定资产投资保持适度规模。晋长高速公路、晋陵一级公路竣工通车，端润一级公路路基和路面铺装基本完成，晋济高速公路开工建设。张峰水库正式开工。晋城污水处理厂进厂总干渠完工，厂区土建施工和设备安装全面展开。晋城垃圾处理厂开工建设。晋城煤层气利用项目前期进展顺利。

　　改革开放取得新的成效。兰花集团积极增资扩股，资本结构进一步优化。天脊晋城化工公司进入上市辅导期。太印、气门两个企业破产结束。乡村集体企业改制继续推进。积极组团参加厦洽会、广交会、东博会、巴西出口商品展销会等招商贸易活动，加强了同国内外的经济技术合作与交流。全市全口径外贸进出口总额完成1.28亿美元，其中自营进出口达到4552万美元，协议利用外资5185万美元，同比分别增长29.8%、48.4%和1.77倍。开发区经济保持强劲发展，财政收入连续第三年实现翻番。富士康晋城科技工业园项目取得实质性进展，前期工作基本结束。

　　社会保障体系进一步完善。“两个确保”成果得到巩固。安置下岗失业人员再就业5512人，城镇登记失业率1.7%，低于省控目标2.8个百分点。农村剩余劳动力转移7万人。发放城市低保金2149万元，受保障人数达到3.1万人，做到了应保尽保；发放农村低保金902万元，受保障人数达到4.3万人。农村新型合作医疗试点工作进展顺利，泽州县参保人数占到农村人口总数的83%。社会救助体系初步形成，帮贫济困活动深入开展，有4.5万多灾民得到救助。

　　各项社会事业全面进步。实施各类科技项目84个，连续第四年被评为“全国科技进步先进市”。继续提高“普九”质量，建成标准化寄宿制小学106所，改造农村中小学危房93所。公共卫生事业进一步发展，市疾控中心投入使用，市传染病医院完成主体工程。文化、体育事业加快发展，成功举办了全国游泳冠军赛暨2004奥运选拔赛、国际围棋名人战、“金镜头”国际摄影颁奖典礼、华夏柳氏宗亲寻根祭祖暨柳宗元文化研讨会等大型活动。人口、资源、环境工作扎实有效。国防动员、双拥、双服务和人防工作得到加强。广播电视、档案史志、外事侨务、民族宗教、妇女儿童、老龄、残疾人等其它各项社会事业也都取得了新的成绩。

　　精神文明和民主法制建设成效显著。多渠道、多形式加强公民道德和精神文明教育，市民素质不断提高。强化社会治安综合治理，深入开展严打整治专项斗争，社会治安状况进一步好转。加强基层基础工作，积极预防、疏导和化解社会矛盾，建成高标准“民主法治示范村”33个。各级政府注重转变职能，改进作风，依法行政水平进一步提高。高度重视人大代表建议和政协委员提案办理工作，全年办理人大代表建议215件，政协委员提案202件。

　　各位代表，过去一年的成绩来之不易。这是省委、省政府和市委正确领导的结果，是市人大、市政协大力支持、有效监督的结果，是全市人民开拓进取、奋力拼搏的结果，是驻市部队、各界人士积极参与的结果。在此，我代表市人民政府，向辛勤工作在各行各业的全市人民，向关心帮助地方工作的驻市部队和武警官兵，向给予政府工作大力支持的社会各界人士，表示崇高的敬意和衷心的感谢！



　　各位代表，过去一年取得的成绩令人鼓舞，但我们也清醒地看到，前进的道路上还有不少矛盾和问题。主要表现在：煤炭产业比重过大，经济结构的深层次矛盾还比较突出；企业产权改革还不到位，自主创新能力还不够强；经济增长方式还比较粗放，环境保护、安全生产压力较大；农民增收的长效机制尚未建立，统筹城乡发展任务艰巨；优质教育和卫生资源供不应求，上学难、看病难等关系人民群众切身利益的问题还没有得到很好解决；政府社会管理和公共服务职能还比较薄弱，一些部门和单位服务意识不强，工作效率不高。对于这些问题，在今后的工作中，我们必须高度重视并认真加以解决。



　　二、2005年政府工作的总体要求和预期目标

　　2005年是全面实现“十五”目标、为“十一五”发展奠定基础的重要一年,又适逢我市建市20周年。今年我们面临的发展环境仍然是机遇和挑战并存。全国经济仍处于新一轮周期的上升阶段，中部崛起战略开始实施，有利于我市发挥资源优势,加快发展步伐；东部经济发达地区加速产业升级，有利于我们承接产业转移，打造产业集群；周边地区你追我赶，发展态势咄咄逼人，对我们既是压力也是动力；经济发展的软环境仍需改善，电力、运力和资金等硬约束依然存在，这都增加了争先发展的难度。面对新的形势，我们不发展不行，不加快发展不行，不科学发展更不行，必须进一步强化危机意识、忧患意识、机遇意识、创新意识和发展意识，以强烈的历史责任感和时代紧迫感，解放思想，抢抓机遇，坚定信心，迎难而上，以更宽的视野、更大的魄力、更实的措施做好今年的工作。

　　今年我市政府工作的总体要求是：以邓小平理论和“三个代表”重要思想为指导，认真贯彻十六大和十六届三中、四中全会精神，进一步树立和落实科学发展观，坚持抢抓机遇、争先发展，坚持以人为本、协调发展，紧紧围绕“四市”建设目标，把握好加快经济结构调整、转变经济增长方式两个关键问题，更加注重解决经济社会发展中的突出矛盾，更加注重解决关系人民群众利益的突出问题，更加注重处理好改革发展稳定的关系，保持生产总值、财政收入、城镇居民人均可支配收入和农民人均纯收入较快增长，努力构建和谐晋城，实现经济社会全面协调可持续发展，为启动“十一五”规划，把晋城建设成为经济发达、人民富裕、生态良好、社会和谐的新兴城市奠定坚实基础。概括起来讲，今年政府工作的重点，就是要围绕“一个目标”，把握“两个关键”，落实“三个注重”，保持“四个增长”。

　　——围绕“一个目标”，就是要围绕市委、市政府提出的建设经济强市、园林城市、一流文明城市和优秀旅游城市的目标，这个目标符合晋城实际，符合科学发展观和构建和谐社会的要求，必须紧紧抓在手上，一抓到底。

　　——把握“两个关键”，就是要把握好加快经济结构调整、转变经济增长方式这两个关键问题，这是今年及今后一个时期解决我市经济社会发展深层次矛盾的切入点。把握“两个关键”，要求我们站在树立和落实科学发展观的高度，针对我市产业结构单一、增长方式粗放的问题，找出主要矛盾，抓住关键症结，理清发展思路，实施项目带动，下大力气把产业做强，把产品做优。

　　——落实“三个注重”，就是要更加注重解决经济社会发展中的突出矛盾、更加注重解决关系人民群众利益的突出问题、更加注重处理好改革发展稳定的关系，这是构建社会主义和谐社会的出发点。落实“三个注重”，要求我们把以人为本的执政理念贯穿于经济社会发展全局，融会到政府工作的各个方面，以发展求和谐，以和谐促发展。

　　——保持“四个增长”，就是要保持生产总值、财政收入、城镇居民人均可支配收入和农民人均纯收入较快增长，这是我市实现争先发展的归宿点。生产总值主要体现经济总量，财政收入主要体现经济增长质量，城镇居民人均可支配收入和农民人均纯收入主要体现人民群众从发展中得到的实惠。保持“四个增长”，要求我们紧紧抓住战略机遇期，坚持以经济建设为中心，优化发展环境，营造发展氛围，在保持经济总量快速增长的同时，使财政的蛋糕大起来，使群众的钱包鼓起来。



　　根据上述总体要求和工作重点，今年全市经济社会发展的主要预期目标是：生产总值达到285亿元，增长12%；财政总收入达到50.8亿元，增长17.9%。其中，一般预算收入达到15.9亿元，增长15.3%；固定资产投资达到128.5亿元，增长20%；社会消费品零售总额达到67亿元，增长12%；外贸系统进出口总额达到5235万美元，增长15%；城镇居民人均可支配收入达到8900元，增长10%；农民人均纯收入达到3540元，增长8%；居民消费价格指数103%；人口自然增长率控制在3.5‰以内；城镇登记失业率控制在4.5%以内；市区空气质量二级以上天数达到210天。

　　确定这样的预期目标，综合考虑了各方面因素，体现了科学发展观的要求和全市人民加快发展的愿望，是符合我市实际的。只要我们集中民智，发挥民力，只要我们众志成城，迎难而上，只要我们开拓创新，奋勇争先，就一定能够抓住发展中的机遇，就一定能够克服前进中的困难,圆满完成今年的各项目标，并力争发展得更快更好。对此，我们充满必胜的信心。

　　三、紧紧扭住经济建设不放松，谋求晋城发展新跨越

　　发展是硬道理，加快发展更是硬道理。面对全国各地百舸争流的发展态势，我们不进则退，慢进也是退。必须把科学发展观贯穿于经济工作的各个方面，统筹兼顾，扎实工作，突出重点，突破难点，促进全市经济更快更好地发展。



　　（一）在结构调整上求突破，努力构筑产业竞争新优势

　　提升优化产业结构，转变经济增长方式，是一项长期而艰巨的战略任务，不会一蹴而就，不可一日不抓。当前，要抓住煤炭市场看好的有利时机，按照 “创优发展环境，大上调产项目，发挥比较优势，做强新兴龙头”的调产思路，充分发挥晋城独特的资源和区位优势，继续推进“四大基地”、“八大产业”建设，全力实施100个重点煤炭企业和100个重点非煤企业的“双百工程”，尽快把我市建设成为全国重要的新型能源和煤化工基地。

　　坚持用高新技术和先进适用技术改造提升传统产业。煤炭产业要继续实施“关小改中建大”战略，积极推进资源整合和集约经营，大力提高生产集中度和装备水平。在抓好9万吨以下小煤矿关闭的同时，加快伯方、唐安、南阳等16座骨干矿井改造,大宁、王坡等骨干矿井要按期建成投产，抓紧东大、玉溪等新建矿井的前期工作。抓好采煤方式改革，年内所有具备条件的矿井全部完成壁式工作面改造。推进煤炭加工转化，延伸煤炭产业链条。突出抓好100个重点煤炭企业建设，保安全，增效益，谋转化，培育财政收入支柱。冶炼、铸造产业要注重抓好大容积高炉的生产运营管理，确保达产达效。以开发区国际铸造园区和泫氏、金工、群英等中高档铸造项目为重点，继续改善装备，改进工艺，扩大规模，力争铸件产量突破100万吨，其中中高档铸件达到50万吨。积极依托铸造优势发展零部件加工业，逐步实现由“晋城铸造”向“晋城制造”的跨越。重视科技进步，加大技改力度，对今年确定的40个重点工业技改项目，要跟踪服务，加强指导，确保顺利完成。

　　以煤化工和电力为重点，努力培育新兴优势产业。我市发展以化肥为主的煤化工产业具有得天独厚的资源、技术和管理优势，应该也必须作为新的支柱产业来抓。今年，要加快天脊“4060”、晋丰“3652”和兰花化工“813”等尿素项目建设，确保年底投产；做好阳城田悦“1830”、煤化工公司二期“2440”和沁水天和化工“6080”一期等尿素项目的前期工作，力争今年开工建设。同时，要努力延伸煤化工产业链条，改善产品结构，提高产品附加值，努力把我市建设成为国内最大的综合性煤化工基地。今年，要抓好高平丹峰30万吨甲醇一期项目建设，确保在年内建成投产；做好天脊晋城化工20万吨甲醇、1.2万吨三聚氰胺项目和兰花集团150万吨甲醇、100万吨二甲醚项目前期工作，确保按时开工建设。电力产业是我市又一个具备发展潜力的优势产业，要加大工作力度，加快电力项目推进步伐。阳电二期项目后天就要正式奠基开工了，这是我市今年电力建设的重中之重。对国投华实晋城热电厂和沁水（鲁能）电厂两个项目，要全力做好前期工作，力争早日开工建设。建材、丝麻、饮品等产业也都要加速资源整合，加快技术进步，培植新的优势，尽快发展壮大。同时，要大力培育发展新材料、生物制药、电子信息等新兴产业，大力实施名牌战略，提高经济增长质量。突出抓好100个重点非煤企业建设，扩规模，上水平，创名牌，培植新的经济增长点。

　　重视发挥好工业园区的产业集聚和辐射带动作用。发达地区的成功实践证明，园区经济对一个地区的经济发展具有重要的产业集聚和辐射带动作用。我市的工业园区起步较晚，必须下大决心，使大气力，精心运筹，科学规划，集中力量办好晋城经济技术开发区和阳城建瓷、巴公化工、高平庞村铸造等重点工业园区。要采取切实有效的措施，理顺管理体制，优化发展环境，加快基础设施建设，扩大招商引资数量，提高招商引资质量。要按照土地集约化经营、产业链条式延伸、企业集群式组合、资源循环式利用的要求，着力提升开发区和其它园区的开发水平，吸引工业项目向开发区和工业园区集中，打造企业集群和产业集群。特别是晋城经济技术开发区必须注重招大商、引大资、上大项目，尽快成长为全市经济的重要增长极。即将在我市开发区建设的富士康晋城科技工业园，以生产光通讯产品和精密零组件为主，总投资5亿美元，占地1000余亩，可吸纳2.5—3万人就业，年产值150—200亿元人民币。该项目的建设，对于我市优化产业结构、提高经济增长质量、增强综合竞争能力具有重大的战略意义，全市上下要进一步统一思想，抓住机遇，创优环境，强化服务，把富士康晋城科技工业园建设放在非常重要的位置抓紧抓好。

　　加强资源和环境保护，推进可持续发展。要立足我市的资源、环境和产业实际，从节约资源中求生存，从保护环境中求发展。要积极探索循环经济之路，推进资源节约、综合利用和清洁生产，推行洁净煤技术，抓好煤炭等资源型初级产品的加工转化，发展煤气发电和瓦斯发电，延伸资源型产业链条，推动资源型城市转型。以创建省级环保模范城为目标，加强城乡环境治理，严格执行排污许可证制度，加强新建项目环境管理，坚决取缔排污不达标的“土、小”企业，突出抓好对城市规划区和城乡结合部的环境监管，加快市区重点工业污染企业的关停、搬迁步伐，抓紧建设陵川、沁水2个国家级生态示范区，完成2个省级环境优美乡镇和12个市级环境优美村的创建工作。



　　积极发展县域特色经济。要鼓励和支持各县(市、区)立足当地实际，把握自身优势，找准发展定位，突出发展重点，培育壮大各具特色的支柱产业。要通过统筹规划，分类指导，优势互补，共同发展，逐步解决县域经济发展不平衡的问题，增强全市整体实力。各县(市、区)在全省综合经济实力的排位要力争前移，工业增加值、非公有制经济增加值占县域生产总值的比重要有明显提高，鼓励经济实力强、发展速度快的地方向全省十强县及全国百强县目标冲刺。



　　积极发展壮大旅游产业。以创建中国优秀旅游城市为目标, 加大投资力度，搞好景区硬件建设，加快旅游区域联合步伐，打造“山西风光·晋城最美”旅游品牌。以“三条线路，五大景区”为重点，积极开展“旅游景区建设年”活动，提升景区品味，促进全市旅游景区上档升级。皇城相府要争创国家5A级景区，王莽岭、珏山、山里泉等景区要争创国家4A级景区。以全国、全省红色旅游线路促销为契机，加大旅游促销力度，积极开拓客源市场，推动旅游产业快速发展，真正把旅游资源优势转化为经济优势和产业优势。积极做好各项准备工作，力争今年通过创建中国优秀旅游城市验收。

　　全力抓好基础设施建设，培植发展后劲。全面完成综合馆、体育场、“两河”治理、“城中村”改造4个扫尾项目。抓好张峰水库、晋城至济源高速公路、市污水处理厂、市垃圾处理厂、市技校综合教学楼5个续建工程。新开工建设晋城煤层气利用项目、阳城至垣曲二级公路、阳城电厂二期工程、晋城500千伏输变电项目等4个重点工程。同时，要做好市图书馆、晋城至侯马高速公路、高平至新乡高速公路、太焦至侯月铁路连接线等重点项目的前期工作。

　　（二）在解决“三农”问题上求突破，努力促进城乡经济良性互动

　　要毫不动摇地把解决“三农”问题作为重中之重，以促进农民增收为核心，跳出“三农”抓“三农”，打破城乡二元结构，加快建立以工促农、以城带乡的反哺机制，认真贯彻“多予少取放活”的方针，着力提高农村经济整体实力，提高农业综合生产能力和市场竞争能力，提高农民收入水平。

　　加大农业结构调整力度。充分发挥比较优势，推进农产品合理布局，加快建设优质粮食生产基地和畜牧、蚕桑、中药材、蔬菜、干鲜果、小杂粮6大特色产业，突出抓好以规模养殖为重点的畜牧业。加快农业标准化建设步伐，积极开展无公害、绿色和有机农产品质量认证，发展品牌农业。大力发展农村二、三产业，促进农民向非农产业转移，拓宽农民增收渠道。集中培植一批经营机制好、市场前景广、产品竞争力强的农业产业化企业，发挥其龙头带动作用，扩张原料基地，拉长产业链条。积极发展农村中介组织和经纪人队伍，提高农民走向市场的组织化程度。加强农村信息体系、市场体系和科技体系建设，为农民增收提供有效服务。

　　加大农村和农业基础设施建设力度。农村和农业基础设施落后是农民增收难的一个重要原因。推进城乡经济良性互动，必须在农村和农业基础设施建设上下大功夫。要抓好4个县城、10个重点镇和200个中心村建设，完善基础设施和公共服务设施，加速城镇化进程，增强城镇吸纳劳动力就业的能力，促进农民向城镇集中。抓好农村道路、电力、通讯等设施建设，注重“村村通”公路维护管理，改善农村用电和通讯条件。从今年开始，要结合“村村通”工程，分期分批实现村村通客运班车。抓好水利设施、小流域治理、中低产田改造等农田基本建设，新发展农业节水面积3万亩，完成水保初治面积16万亩，改造中低产田18万亩。抓好农村生态建设，完成植树造林9.7万亩,乡村公路绿化1500公里，建设30个高标准小康生态绿化村。

　　加大农村扶贫开发和奔小康力度。以整村搬迁为重点，继续搞好扶贫移民开发。对原晋城、高平、阳城、陵川、沁水5个抗日政府所在地要加大扶持力度，尽快改善“老区中老区”的生产生活条件。今年要完成2000人的移民搬迁和9000人的稳定脱贫。农村小康建设要更加注重发展社会事业，促进全面进步。今年要建成100个宽裕型小康村、6个宽裕型小康乡（镇），城区要整体建成宽裕型小康区。积极推进泽州县农村现代化建设试点工作。



　　加大农业和农村工作政策落实力度。继续落实农村税费改革政策，从今年开始，在全市全部取消农业税及其附加。落实粮食直补、良种补贴和购置大型农机具补贴政策，增加粮农收入。完善农民负担监督管理机制，抓好涉农收费专项治理，继续减轻农民负担。坚持和完善农村土地承包制度，规范农村土地承包经营权流转程序和行为，改革和完善征地补偿制度，切实维护农民合法权益。深化粮食流通体制改革，推进粮食产业化经营。实行最严格的耕地保护制度，确保耕地动态平衡。

　　（三）在城市建设和管理上求突破，努力创造良好的人居环境

　　今年是我市建市20周年，20岁的晋城正值青春年华，正好扬帆破浪。建市以来，中心城市建设以拉开框架、拓展规模、美化环境、整治市容为重点，收到了明显成效。适应区域经济和城市发展的需要，今后城市建设的重点要逐步转向强化基础、完善功能、挖掘内涵、塑造特色，以进一步提升城市品位，创造良好的人居环境。

　　坚定不移地走经营城市之路。管理和配置好城市资源，用活包括城市土地在内的所有资产，加大土地收购储备力度，垄断土地一级市场，搞活土地二级市场，严格执行经营性用地“招、拍、挂”制度，不断提升城市资源价值。公开拍卖户外广告发布权和城市道路、桥梁、广场、游园冠名权，提高城市基础设施自我积累、自我发展的能力。深化城市公用事业改革，放开市政公用市场，采取统一规划、市场运作的方式，鼓励社会资本参与公用设施建设和经营，探索建立城市公用设施建设的多元化投入模式和市场化经营模式。

　　精心实施城市建设“六大工程”。一是“蓝天工程”。通过关停污染企业、控制扬尘污染、禁用散煤、扩大集中供热等措施，确保市区空气质量二级以上天数达到210天以上；二是“畅通工程”。完善道路基础设施，合理控制交通流量，加强停车场（点）管理，优先发展公共交通，解决好“瓶颈”路段高峰期交通拥堵问题。开工建设白水街道路，改善安置小区基础设施；三是“绿化工程”。新建绿地700亩，完成8个小区和57个庭院绿化达标，完成泽州南路、黄华街南段、东环路中段、前进路北段4条道路绿化。市区绿化覆盖率、公共绿地率、人均公共绿地面积要分别达到35%、30%和 9平方米以上，为创建国家级园林城市和中国优秀旅游城市创造条件；四是“美化、亮化工程”。继续完善泽州路、风台街绿化带亮化工程，提高泽州路、风台街美化亮化标准，规范亮化设施管理；五是“净化工程”。市污水处理厂、晋煤集团污水处理厂和巴公工业园区废水处理厂年内要投入使用，市垃圾处理厂要抓紧建设；六是“碧水工程”。巩固完善“两河”和白水河治理成效，形成15万平方米的水面。加快西河缓洪库和东焦河水库建设，增强城市防洪功能。在抓好以上六大工程的同时，要全面完成黄华街西区“城中村”改造工程，确保1530户居民全部回迁。

　　进一步提高城市管理水平。要坚持建管并重，标本兼治，构建城市管理长效机制。按照职权法定、权责一致的原则，规范行政执法主体，建立协调机制，继续完善和推进城市管理综合行政执法。加大市容环境整治力度，重点整治市区乱停乱放、乱贴乱画、乱摆摊点、占道经营、无证经营等群众反映强烈的问题。城市管理要向背街小巷延伸，消除管理盲区和盲点。引导广大市民增强城市意识，提高文明素质，树立文明形象，营造热爱晋城、关心晋城、建设晋城的社会氛围，全方位提高城市文明程度。

　　（四）在深化改革、扩大开放上求突破，努力增强经济发展的动力和活力

　　深化改革、扩大开放是推动经济发展永不衰竭的强大动力。我们必须敢于破除那些不利于发展的条条框框，以所有制结构调整为切入点，大胆进行制度创新、体制创新，努力推动改革开放迈出新的步伐。

　　深化国有企业改革。对所有竞争领域的中小型国有企业，进行彻底的产权制度改革。推进市属国有企业整合，完成太印、气门两厂破产后的重组和晋钢破产关闭工作，抓紧进行绿洲大麻和凤凰织品两公司的同步改制与重组。以“两个置换”为中心，今明两年要基本完成市、县两级国有工商企业的改革工作，用三年时间剥离企业办社会职能。大力发展混合所有制经济，实现投资主体多元化。积极推动煤炭企业走集团化、规模化的路子，完成晋城无烟煤集团的组建。抓好天脊晋城化工公司上市准备工作。积极探索国有资产监管和经营的有效形式，确保国有资产保值增值。

　　推进乡村集体企业改革。以产权为核心，进一步加快乡村集体企业股份制改造或民营化。对有一定规模和发展潜力的企业，进行股份制改造，完善企业组织结构；对规模小、效益差的企业一律进行拍卖出售；对现有停产、倒闭企业通过重组的办法，盘活存量资产。按照资源资产化管理、企业股份化改造、区域集团化重组的工作思路，抓好乡村集体煤炭企业改革，努力在推行资源有偿使用、提高产业集中度和安全生产水平上取得突破。

　　放手发展民营经济。要充分认识民营经济在促进经济增长、扩大就业和繁荣市场等方面的重要作用，在降低门槛、加强服务、积极扶持、依法发展等方面大做文章，为民营经济创造宽松有序的发展环境。要进一步放心、放胆、放手，对民营经济在市场主体地位、市场准入和资源配置上切实做到一视同仁，努力形成既有顶天立地的大型民营企业、又有铺天盖地的中小民营企业的发展势头，提高民营经济在全市经济总量中的比重。对重点建设的83个项目，要从技术、人才、管理、服务等方面加强扶持，年底力争投产运行65个，亿元以上营业收入企业达到18个。全市民营经济增加值突破100亿元，上交税金突破14亿元，分别增长25%以上。

　　深化财政体制和投资体制改革。加快构建公共财政框架，优化财政支出结构，加大对农业、教育、科技等领域的保障力度。进一步深化部门预算、国库集中收付、政府采购和“收支两条线”管理改革。进一步拓展财源，形成财政收入持续快速增长机制。强化税收征管，坚持依法治税。规范投资项目的审批、核准和备案，落实企业投资自主权。规范政府投资行为，财政资金要用于提供公共产品、举办公益事业和提供公共服务，办那些公众需要办而企业、社会、民间不愿办的事。建立政府投资项目综合考评和责任追究制度。增强政府投资的导向和带动作用，尽快形成投资主体多元化、资金来源社会化、投资方向多样化的固定资产投资格局。

　　加快市场体系建设。推进流通活市战略，深化流通体制改革，加快物流平台建设，在重点商业领域广泛拓展连锁经营、物流配送、电子商务等新型流通业态，增强服务功能，提高竞争能力，全市新型业态销售额要达到社会消费品零售总额的30%左右。顺应消费结构升级趋势，着力培育和壮大文化娱乐、教育培训、体育健身、卫生保健、社区服务、住房、汽车等新的消费热点。挖掘农村消费潜力，培育农村消费市场。积极发展资本、土地、技术和劳动力市场，建立和规范产权交易市场，促进各种要素合理流动。加大金融创新力度，推进农村信用社改革，加强银企合作，改善金融服务，全力支持地方经济建设。继续整顿和规范市场经济秩序，深入开展食品安全、知识产权保护、打击商业欺诈等专项治理行动，营造安全放心的市场消费环境。

　　继续扩大对外开放。紧紧抓住国内外产业梯度转移、资本加速流动的难得机遇，立足国际国内两个市场、两种资源，积极推进全方位开放。实施“走出去，引进来”战略，突出企业的主体作用，组织好各项招商贸易活动。利用市综合馆即将投入使用的条件，从承办小型展会入手，积极探索发展会展经济，为促进合作与交流提供平台。创造更为宽松的投资环境，用优质、高效、廉洁的服务，积极引进外资，引进港、澳、台投资，特别是要进一步发挥优势，打好台资牌，借船出海，借鸡生蛋。加快商务管理体制改革，创新引资方式，突出专业招商，主动承接、吸纳国内发达地区、知名企业的产业转移和辐射。加强与周边地区的经济技术合作与交流，特别是要主动出击，积极接轨以郑州为中心的中原城市群，融入中原经济圈。积极调整出口产品结构，加大高档铸件、汽车配件、机电配件、玻璃器皿、精细化工产品、纺织服装的出口比重。今年，全市全口径外贸进出口总额要达到1.5亿美元，实际利用外资要突破5000万美元。

　　四、切实注重以人为本，努力构建和谐晋城

　　在加快经济发展的同时，必须把构建和谐社会放在更加重要的位置，寻求效率与公平的最佳结合，切实解决好与人民群众利益密切相关的问题，促进经济与社会、人与自然、社会各阶层和不同利益群体之间的和谐。

　　（一）突出加强精神文明建设和教育、科技工作

　　加强精神文明建设。以纪念抗战胜利60周年和晋城建市20周年为契机，大力弘扬以爱国主义为核心的民族精神和以改革创新为核心的时代精神。加强公民思想道德建设，移风易俗，倡导科学、健康、文明的生活方式。特别是要注重未成年人思想道德建设工作，努力营造有利于未成年人健康成长的社会环境。以创建全国文明城市先进市为目标，广泛开展“做文明晋城人，建文明晋城市”活动，加大中心城市、县城、生态型小城镇、宽裕型小康村“四位一体”的城乡连片创建力度，努力提高文明县城、文明村镇、文明行业、文明单位的创建水平，夯实文明城市的创建基础。

　　坚持优先发展教育事业。启动全市教育攻坚。动员社会力量，改善办学条件，整合教育资源，提升义务教育。加快建设农村标准化寄宿制学校，对农村小学实行高段合并；整合市区和城镇中小学教育资源，缓解市区中小学班容量过大和入学难问题。实行民办公助，扩大高中规模，提高教学质量，满足就学需求，努力缓解高中上学难问题，提高初中毕业生升学率。发展职业教育，培养技术人才，提高劳动技能，增强就业本领。抓好市职业技术学院建设和管理，努力提高办学质量。完善成人教育培训网络。积极推进教育体制改革，大力提倡社会办学，动员和鼓励社会各界兴办教育或捐资兴办教育。在落实对农村义务教育阶段家庭经济困难学生实行“两免一补”政策的基础上，免除义务教育阶段所有农村家庭及城镇低保家庭学生的杂费，确保他们顺利完成九年义务教育。

　　积极做好科技和人才工作。继续实施《晋城科技行动方略》，打造科技服务平台，重点抓好实用科技推广普及工作。深化市直科研机构改革，加强和大专院校、科研院所的合作，立足我市产业发展需要，做好科学技术向生产力的转化工作，推动科技创新。充分挖掘人才作为第一资源的巨大潜能，努力做到人才结构调整与产业结构调整相同步、人才资源开发与经济社会发展相协调。不断拓宽人才引进通道，树立“天下人才，为我所用”、“不求常住，但求常来”、“不求所有，但求所用”的观念，完善吸引人才、留住人才、利用人才的政策措施，在全社会营造尊重人才的良好氛围。

　　加快发展文化、卫生、体育等各项社会事业。大力发展文化事业和文化产业，繁荣农村文化，培育市民文化，活跃校园文化、企业文化和广场文化。举办好第二届“金镜头”国际摄影颁奖活动。加大文化基础设施建设力度，特别是要以纪念赵树理诞辰100周年为契机，积极筹建赵树理文学馆，拍摄、排演《赵树理》电视剧和戏剧，打造特色文化品牌。加强农村卫生工作，提高农村医疗卫生队伍素质。推进城镇医疗卫生体制改革，引导和扶持民营医疗机构发展，加快社区卫生服务体系建设。完善公共卫生和疾病防控体系，提高突发公共卫生事件处置能力和城乡公共卫生水平，努力创建国家级卫生城市。广泛开展全民健身活动，办好市运会，备战好省运会，精心组织好亚洲青少年花样游泳锦标赛等大型体育比赛。做好人口和计划生育工作，稳定低生育水平，提高出生人口质量。重视国防教育，深入开展双拥和双服务活动，加强人防工作。继续做好广播电视、民族宗教、妇女儿童、档案史志、外事侨务、气象、统计、地震、老龄、残疾人等各项工作。

　　（二）突出关注民生问题

　　做好就业和再就业工作。落实财政投入、小额贷款、免费培训、税费减免等各项促进就业政策，强化就业服务，改善就业环境。通过发展劳动密集型产业、就业容量大的民营经济和中小企业,扩大劳务输出，购买公益性岗位等途径增加就业，今年新增城镇就业岗位2.4万个，输出和转移农村剩余劳动力7万人。抓好下岗失业人员再就业技能培训，推广职业资格认证制度，鼓励企业优先录用本地下岗人员，今年安置下岗失业人员再就业4080人，其中“4050”人员870人。加强劳动力市场管理，严格执行企业最低工资制度，加强劳动保护，探索保障农民工工资发放的有效办法，依法维护劳动者合法权益。关注新失业群体，重视高校毕业生、复转军人的就业指导和服务。 

　　进一步完善社会保障体系。全面推进养老、失业、医疗、工伤和生育保险制度建设，强化社会保险基金的征缴，确保按时、足额发放。抓好国有企业下岗职工基本生活保障向失业保险并轨工作，维护职工利益。进一步扩大社会保险覆盖面，积极提高非公有制企业、个体工商户和灵活就业人员参保率。继续巩固和完善农村最低生活保障制度，扩大覆盖范围。高度重视城市化进程中失地农民的保障问题，使失地农民安居乐业。健全城乡社会帮扶救助体系，真正关心弱势群体，帮助特殊困难群众解决基本生活和子女上学、看病就医等方面的实际问题。

　　集中力量办好20件实事。今年，要继续为农民办好10件实事：（1）全部免征农业税及其附加；（2）抓好农村饮水解困，解决6万人饮水困难；（3）资助规模养殖业，确保农民人均畜牧业纯收入比上年增长10%以上；（4）构建动物防疫体系，建设15个畜牧兽医中心站；（5）完成山庄窝铺整村移民搬迁2000人；（6）完成10万人（次）农民素质培训；（7）实施农村助学工程，加快建设农村标准化寄宿制学校，全部免除义务教育阶段农村学生杂费2400万元；（8）启动城区新型农村合作医疗工作；（9）继续实施偏远贫困地区电视覆盖工程；（10）巩固和完善农村最低生活保障制度。为市民办好10件实事：（1）继续实施蓝天工程；（2）建设四期集中供热工程，新增供热能力80万平方米；（3）加大民办公助高中建设力度，缓解市区高中入学难问题；（4）继续实施道路畅通工程；（5）继续实施市区绿化工程，新增绿地46公顷；（6）建设城市燃气补充气源工程，提高燃气覆盖率；（7）建设市区低洼地防洪工程，增强市区防洪能力；（8）对旧城区总长4.7万米的供水管网进行改造；（9）建设20座星级公厕和垃圾中转站，改善城市卫生设施；（10）完成36万平方米的安康居住工程，缓解城镇中低收入家庭的住房困难。

　　（三）突出抓好社会稳定和安全生产



　　加强民主法制建设。积极推进普法宣传，采取法制讲座、送法下乡、街头普法、法进社区等多种形式，不断提高各级领导干部和全市公民的法律素质，营造全社会遵法守法、依法维权的良好环境。继续推进“民主法治示范村”建设，积极探索农村学法普法和依法治理新途径。认真做好第七届村民委员会换届工作，继续强化城市社区建设，进一步推进基层民主。

　　全力维护社会稳定。认真落实信访工作责任制，抓好新的《信访条例》的学习宣传和贯彻落实，切实加强县（市、区）、乡（镇）、村三级信访工作，把信访问题解决在基层，解决在萌芽。加大对不理民事者的责任追究力度。规范信访秩序，依法打击违法行为。健全正确处理人民内部矛盾的工作机制，深入开展矛盾纠纷排查调处工作。充分发挥人民调解、司法调解、行政调解的作用，多方面、多层次化解矛盾纠纷，切实处理好在农村土地征用、城镇房屋拆迁、国有企业改制、乡村集体煤矿非法承包过程中发生的涉及群众切身利益的问题。进一步强化社会治安综合治理，推进社会治安防控体系建设,形成覆盖城乡的治安防控网络。深入开展“严打”整治斗争，严厉打击 “法轮功”等邪教组织和境外敌对势力的渗透破坏活动，严厉打击严重刑事犯罪尤其是多发性侵财犯罪和严重暴力犯罪，严厉打击破坏社会主义市场经济秩序的违法犯罪。深入开展“扫黄打非”和禁毒斗争，扫除各种社会丑恶现象。



　　切实抓好安全生产。进一步落实安全生产责任制，加大安全生产执法力度，严格安全生产责任追究。特别是要强化对安全工作重点行业的监管，加大安全质量标准化矿井建设力度，加大安全资金投入力度，做好矿井安全监控系统升级工作，加强高瓦斯、高突出、高危险矿井的管理，严厉打击非法生产，坚决控制超能力生产，对所有改采煤矿强制配备专业工程技术人员，对所有煤矿配备驻矿安监员。高度重视道路交通安全工作，重点抓好宣传教育和危险路段整治，严防重特大事故的发生。同时，要下大力抓好特种设备、烟花爆竹、危险化学品、非煤矿山、公众聚集场所的专项安全整治。今年，煤矿百万吨死亡率要控制在0.9以下，交通事故起数和死亡人数要低于全国、全省平均水平。

　　五、加强政府自身建设，进一步提高行政能力

　　面对改革开放和现代化建设的新形势、新任务，各级政府必须牢固树立和贯彻科学发展观和正确政绩观，按照保持共产党员先进性教育活动的要求，不断提高行政能力，努力建设人民满意的政府。

　　（一）推进思想解放，建设活力政府。惟有思想大解放，才有事业大发展。适应我市新一轮大发展、大改革、大开放的需要，从政府自身抓起，推进全市上下干部群众思想的大解放，推动晋城改革开放和现代化建设的大跨越。特别是在当前机遇与挑战并存、发展面临考验的情况下，不能有任何的摇摆和犹豫，必须高举解放思想的大旗，一门心思抓发展，全力以赴抓建设，保持争先发展、加快发展、有效发展的强劲势头。要更好地学会把中央的大政方针与本地实际紧密结合起来，正确处理好宏观与微观、共性与个性、整体与局部的关系，多从积极的方面考虑问题，少说“不能做”，多想“怎么办”，只要在法律法规框架内，只要符合“三个有利于”标准，就要积极试、大胆干。要进一步解决好工作创新问题，用创新突破思维定势，用创造破解发展难题，用超常规的举措推动超常规的发展。要进一步解决好干部精神状态问题，始终保持昂扬向上、勇于进取的朝气，始终保持奋发有为、敢于争先的锐气，始终保持自加压力、负重奋进的勇气，始终保持百折不挠、永不言败的志气，使政府充满蓬勃生机和旺盛活力。

　　（二）提高行政能力，建设效能政府。面对晋城发展的历史重任，必须坚持把加强行政能力建设作为一项长期的基础性工作，在政府各部门和全体公务人员中大力营造“重学习、重思考、重实践”的浓厚氛围，用邓小平理论和“三个代表”重要思想武装头脑，提高理论素养，开阔世界眼光，培养战略思维，努力提高运用科学发展观指导实践、解决问题的能力。特别是要增强加快发展的能力，正确分析经济形势，深入研究政策措施，深刻认识经济发展的规律性，牢牢把握经济发展的主动权，以争先发展的实际成效检验政府的行政水平。要努力转变政府职能，变无限政府为有限政府，变低效政府为高效政府。令即行，禁即止，保证政令畅通，解决好不作为或乱作为的问题。加强软环境建设，优化政府工作流程，积极发展电子政务，努力提高行政效率。发扬雷厉风行的好作风，克服办事拖拉的坏习惯。

（三）严格依法行政，建设法治政府。认真贯彻实施《依法行政纲要》，严格按照法律规定的权限和程序行使权力、履行职责。坚持权力与责任挂钩、权力与利益脱钩的原则，约束行政权力，做到有权必有责、用权受监督、侵权要赔偿、违法要追究。深化行政执法体制改革，加强行政执法监督，坚决纠正执法过程中的违法乱纪行为。完善公众参与、专家论证和政府决策相结合的决策机制，规范决策程序，强化决策责任，提高决策的科学化、民主化水平。认真贯彻执行《行政许可法》，进一步深化行政审批制度改革，尽量减少审批事项，简化审批程序。对“吃、拿、卡、要”者，毫不留情，绝不手软，坚决给予严肃处理。实行政务公开，自觉接受人大及其常委会的监督，认真执行人大及其常委会的决议、决定，主动向人大及其常委会报告工作。自觉接受政协的民主监督，密切同各民主党派、工商联、无党派人士、社会各界人士和人民团体的联系，认真听取他们的意见和建议。自觉接受广大人民群众和新闻舆论的监督，从群众中汲取智慧和力量。

　　（四）弘扬实干精神，建设务实政府。面对全市人民盼富求强的迫切愿望，我们只有实干才能争先发展，只有实干才能取信于民，只有实干才能让老百姓过上幸福日子。要坚决反对以会议贯彻会议、以文件落实文件的做法，大力精简各类会议和各种文件，严格控制各类检查、评比和达标活动，把广大干部从文山会海和迎来送往中解脱出来，真正把工作的立足点放在真抓实干上，放在务求实效上。始终坚持正确的政绩观，坚持重实际、办实事、求实效的理念，坚持脚踏实地、埋头苦干、干就干成的作风，坚决反对官僚主义、形式主义，坚决反对欺上瞒下、推诿扯皮、弄虚作假的行为，坚决反对只说不做、不推不动、虎头蛇尾的作风。领导干部要率先垂范，扑下身子，深入一线，苦干实干，形成一级抓一级、层层抓落实的局面，真正把工作任务不折不扣地落到实处。

　　（五）恪守执政为民，建设亲民政府。政府的所有干部都来自百姓，政府的一切权力都属于人民。各级政府和广大公务员的一切工作都要以人民群众满意不满意、赞成不赞成、拥护不拥护、答应不答应为衡量标准，真正做到权为民所用、情为民所系、利为民所谋。继承和发扬党的优良传统，切实增强群众观念，做到在思想上尊重群众，在感情上贴近群众，在工作上依靠群众。始终坚持以人为本、关注民生、体恤民情，时刻把群众的冷暖、百姓的愿望放在心上，想人民所想，急人民所急，帮人民所需，当好人民的公仆。始终坚持勤政为民，在经济发展的过程中决不损害群众利益，在经济发展的基础上努力改善群众生活。



　　（六）坚持从严治政，建设廉洁政府。认真开展好保持共产党员先进性教育活动，使广大公务员树立正确的理想信念,保持高尚的道德情操，一要干事、二要干净。坚决贯彻中央、省委和市委廉洁自律的各项规定，建立健全教育、制度、监督并重的惩治和预防腐败体系，认真落实党风廉政建设责任制。各级领导干部要自觉加强党性修养，时刻自重、自省、自警、自励，增强在诱惑面前的自控力，在警示面前的自省力，筑牢拒腐防变的思想防线，始终保持浩然正气。在这里，我代表市政府一班人向全市人民郑重承诺：绝不在行使行政审批权和分配使用财政资金过程中搞权钱交易；绝不利用职权违反规定干预和插手建设工程招标、经营性土地使用权出让、房地产开发与经营等市场经济活动；绝不违反规定收受现金、有价证券和支付凭证；绝不放任、纵容配偶、子女及身边工作人员利用领导干部职权或职务影响以权谋私。请各位人大代表和全市人民监督。

　　编制国民经济和社会发展第十一个五年规划，谋划好全市发展的宏伟蓝图，是今年各级政府的一项重要工作，必须高度重视。要创新规划理念，规范编制程序，加强调查研究，充分发扬民主，注重衔接协调，突出发展重点，切实提高规划的科学性、前瞻性和可操作性。

　　各位代表，站在新的起点，我们深知责任重大，我们充满必胜信心。让我们紧密团结在以胡锦涛同志为总书记的党中央周围，在中共晋城市委的领导下，同心同德，扎实工作，迎难而上，勇往直前，共同创造晋城更加美好的明天！
